# LabKey Server: An open source platform for scientific data integration, analysis and collaboration

**DOI:** 10.1186/1471-2105-12-71

**Published:** 2011-03-09

**Authors:** Elizabeth K Nelson, Britt Piehler, Josh Eckels, Adam Rauch, Matthew Bellew, Peter Hussey, Sarah Ramsay, Cory Nathe, Karl Lum, Kevin Krouse, David Stearns, Brian Connolly, Tom Skillman, Mark Igra

**Affiliations:** 1 LabKey Software, Seattle, WA, 98102, USA; 2 Statistical Center for HIV/AIDS Research & Prevention (SCHARP), Fred Hutchinson Cancer Research Center, Seattle, WA, 98109, USA

## Abstract

**Background:**

Broad-based collaborations are becoming increasingly common among disease researchers. For example, the Global HIV Enterprise has united cross-disciplinary consortia to speed progress towards HIV vaccines through coordinated research across the boundaries of institutions, continents and specialties. New, end-to-end software tools for data and specimen management are necessary to achieve the ambitious goals of such alliances. These tools must enable researchers to organize and integrate heterogeneous data early in the discovery process, standardize processes, gain new insights into pooled data and collaborate securely.

**Results:**

To meet these needs, we enhanced the LabKey Server platform, formerly known as CPAS. This freely available, open source software is maintained by professional engineers who use commercially proven practices for software development and maintenance. Recent enhancements support: (i) Submitting specimens requests across collaborating organizations (ii) Graphically defining new experimental data types, metadata and wizards for data collection (iii) Transitioning experimental results from a multiplicity of spreadsheets to custom tables in a shared database (iv) Securely organizing, integrating, analyzing, visualizing and sharing diverse data types, from clinical records to specimens to complex assays (v) Interacting dynamically with external data sources (vi) Tracking study participants and cohorts over time (vii) Developing custom interfaces using client libraries (viii) Authoring custom visualizations in a built-in R scripting environment.

Diverse research organizations have adopted and adapted LabKey Server, including consortia within the Global HIV Enterprise. Atlas is an installation of LabKey Server that has been tailored to serve these consortia. It is in production use and demonstrates the core capabilities of LabKey Server. Atlas now has over 2,800 active user accounts originating from approximately 36 countries and 350 organizations. It tracks roughly 27,000 assay runs, 860,000 specimen vials and 1,300,000 vial transfers.

**Conclusions:**

Sharing data, analysis tools and infrastructure can speed the efforts of large research consortia by enhancing efficiency and enabling new insights. The Atlas installation of LabKey Server demonstrates the utility of the LabKey platform for collaborative research. Stable, supported builds of LabKey Server are freely available for download at http://www.labkey.org. Documentation and source code are available under the Apache License 2.0.

## Background

To gain insight into complex, variable diseases like HIV, researchers need to bring together many different types of information from varied sources at early stages of research. Software systems that provide secure data integration, analysis and sharing can facilitate collaborative efforts against such diseases; however, existing software has significant limitations. Existing software systems typically do not span the full flow of data through an organization, require commercial licenses, focus on limited data types, provide limited extensibility, or cannot easily be used beyond the organizations that designed them. We developed LabKey Server as an end-to-end, "biology-aware" data integration platform that can be customized to meet the needs of diverse research organizations. The source code is freely available under the non-restrictive Apache License 2.0[1]. The system has been proven in heavy production use and is maintained by a professional development team.

One of the largest installations of LabKey Server is called Atlas. It is managed by the Statistical Center for HIV/AIDS Research and Prevention (SCHARP) at the Fred Hutchinson Cancer Research Center. This installation illustrates the core capabilities of LabKey Server and demonstrates how these capabilities have helped a large organization accelerate and enhance research efforts.

The vast majority of LabKey Server features developed for Atlas are built into the LabKey Server platform and available as part of the open source project. Certain customizations of the Atlas installation are closely tailored to particular projects or studies, so they are not part of the open source project. They are only mentioned here as illustrations of extensibility, and they are noted as such.

Atlas has grown out of SCHARP's efforts to meet the needs of several consortia within the Global HIV Vaccine Enterprise (the Enterprise) [2]. The Enterprise is a virtual coalition of researchers that aims to accelerate progress towards one of the most challenging problems in medicine, the development of HIV vaccines[2-6]. Following the example of the Human Genome Project[7], the Enterprise aims to set common goals, standardize processes and share data and techniques as soon as they are developed. Just like the Human Genome Project, this endeavour requires a massive data integration effort. Unlike the Human Genome Project, but like other large-scale, collaborative efforts against intractable diseases, the Enterprise must integrate a large number of data types. These include results from diverse assays, clinical records and sample information. Though Atlas is not a formal project of the Enterprise itself and has no official endorsement, it is used by a variety of consortia within the Enterprise to accelerate scientific discovery.

### Requirements

Uniting distributed efforts to investigate the biology and the treatment of an evolving disease poses challenges for data management tools. To gain insight into viral/host dynamics, researchers need to bring together diverse types of data (*e.g*., viral loads, specimen records and clinical notes) at all stages of research, even when the data originate from multiple labs and clinics across the globe. Researchers need to be able to see many different data types simultaneously to investigate study participants who have exceptional immune responses, such as elite controllers or rapid progressors. They require the agility to extract lessons from failed trials and move investigations quickly in new directions, or to swiftly scale up their successes. Researchers require tools to support the development, standardization and dissemination of new, improved assay protocols and workflows across organizations. Furthermore, they need to be able to quickly apply new analysis techniques to existing datasets without the assistance of computer programmers. Tools must be capable of handling the quantity and complexity of data generated by high-throughput technologies. As a team, they need to improve the quality, reproducibility and comparability of data through standardization of lab measurements and procedures. Globally distributed teams need to rapidly, securely exchange information and specimens, ideally through a single, unified interface.

### Alternatives

Although existing software tools[8-28] could meet some of the requirements of the Atlas project, none meet all of them in the form of a comprehensive, end-to-end platform available as open source. Some tools have experienced only limited use. A few broad, commercial systems have recently been introduced (*e.g*., Microsoft Amalga Life Sciences[29], Genologics[30], Genedata[31] and Axiope eCAT[32,33]); however, they lack the transparency of open source solutions, so they are not reviewed here. Existing open source tools typically lack key features, such as role-based permissions, document sharing, easy extensibility, specimen requests, observational study management, full-text search, dynamic interaction with external data sources, integration with analysis tools like R, and support for describing arbitrary, complex experimental data types. Table 1 provides an overview of the feature tradeoffs between representative platforms.

**Table 1:** Feature tradeoffs between platforms

	*Platforms*					
*Features*	CAISIS	i2b2	SIMBioMS	ISA	Intermine	LabKey Server
Specimen requests	+	-	-	-	-	+
Role-based permissions	+	+	-	-	+	+
Built-in understanding of clinical study entities (*e.g*., participants and visits)	+	+	+	-	-	+
Management of high-throughput assay results	-	-	+	+	+	+
Wizards for collecting custom metadata for experiments	+	+	+	+	-	+
Result schemas (not just metadata) customizable through graphical interface	+	-	-	-	-	+
Broad range of customizable, scientifically-relevant properties for every column of data	-	-	-	-	-	+
Graphical tools for setting up lookups between tables	+	-	-	-	-	+
Complex queries on experimental results, not just metadata	+	+	-	-	+	+
Libraries to support programmatic data manipulation and user interface creation from external code	-	+	-	-	+	+
Built-in user interface for scripting in R	-	-	-	-	-	+
Dynamic interaction with external data sources	-	-	-	-	-	+

This table compares LabKey Server with a representative sample of open source platforms for data integration. The focus of these platforms ranges from clinical research to high-throughput experiments. Documentation for many platforms is incomplete, so we can provide only a reasonable inference of feature availability.

To our knowledge, no other open source tool provides support for both web-based specimen requisitions and integration of specimen data with complex experimental results. For example, PASSIM[8] (and derivatives SLIMS[34] and SIMBioMS[9]), caTissue[35], ePIMS[36] and BASE[10]) all provide sample provenance tracking, but none of these allow for web-based sample requests. eOncoLIMS[37] supports equipment requests and GNomEx[38] supports experimental work requests, but neither one supports specimen transfers. i2b2[11] has some form of a sample request module (i2b2 - Crimson) under construction, but it has not yet been released. BSI[39] provides sample requisition support but does not provide for integration of sample and experimental data. CAISIS[12,13] is exceptional in providing both specimen requests and deep support for data integration; however, it only supports simple test results entered through online forms, not complex experimental data types.

Many tools allow users to describe and collect custom metadata for experiments (e.g., Addama[14], BASE[10], iLAP[15], SIMBioMS[9], ISA[16]). Several tools (*e.g*., iLAP[15], ISA[16] and SIMBioMS[9]) also provide customizable or domain-standardized wizards for collecting metadata for experiments during data import. Unfortunately, all of these tools store only metadata in their databases, not results. Keeping results out of a database makes perfect sense for exceptionally large result sets (*e.g*., microarray results); however, database import is often desirable for smaller datasets because it allows SQL-based querying. Open source software does not typically provide graphical, run-time tools for describing schemas for arbitrary, complex assay results and then performing advanced queries over both data and metadata.

Furthermore, no other open source platform known to us provides graphical facilities for defining a broad range of customizable, scientifically-relevant properties for any column of data (such as missing value indicators, regular expression validators, default values and lookup relationships). CAISIS[12] allows the definition of a few of these properties (default values, defined vocabularies and collection requirements) for simple lab and clinical results; however, it provides no support for complex experimental data.

Many of the widely known frameworks for data integration (*e.g*., BioMart[40]) are tailored primarily towards working with published data, after results have reached "finished" form, not for integrating evolving data types during the research process[14]. Even among tools targeted towards research data, such as electronic lab notebooks, alteration or addition of data types typically requires database alterations[17]. Tools designed for integrating raw research data often work only with specific data formats (*e.g*., SBEAMS[18], caIntegrator[41] and GenePattern[42]) or support the introduction or extension of data types only when the system is not running (*e.g*., Intermine[19]). Even when tools provide flexibility in defining relationships between tables (*e.g*., Intermine[19]), they typically lack graphical tools for doing so. Such approaches are practical when data types are reasonably static and standard, but not when these types need to evolve quickly, without developer support, as research advances[14].

Many data integration tools (*e.g*., Intermine[19], BioMart[40] and Atlas (unrelated to SCHARP's Atlas)[20]) lack dynamic access to external data sources and require aggregation of all data into a central warehouse. Updates can be challenging when external data sources change[43,44]. Fully decentralized approaches (such as those used by BioMOBY[45]) are not easily amendable to consistent quality control[46].

Open source clinical data management software tools (*e.g*., CAISIS[12,13], OpenClinica[47] and openCDMS[48]) typically lack features necessary for managing both study data and highly dimensional experimental data. For example, they typically lack the ability to collect complex assay data in batches of runs. When open source systems do facilitate integration of both study and experimental data types, they typically support only limited data types or allow only narrow queries. For example, SIMBioMS[9] understands relationships between participants, specimens and experimental results; however, it lacks extensible types, recognizes only particular data file formats, and allows users to filter only on metadata, not to fully query experimental results. i2b2[11] provides more of the querying capabilities desired by SCHARP. However, it requires that all imported data map to a set of fixed schemas, lacks data type extensibility and does not support experimental data management.

A growing number of tools (*e.g*., Atlas (unrelated)[20]) furnish client libraries, but few (e.g., GMOD-DBSF[21] and Intermine[19]) provide APIs both for customizing interfaces and for querying data. Many tools that focus primarily on biological data integration (e.g., BioMart[40], GMOD-DBSF[21], Intermine[19], iLAP[15] and Addama[14]) supply some form of integration with open source analysis tools; however, none known to us provides a built-in, graphical R interface.

### The Atlas Installation of LabKey Server

**Figure 1:**
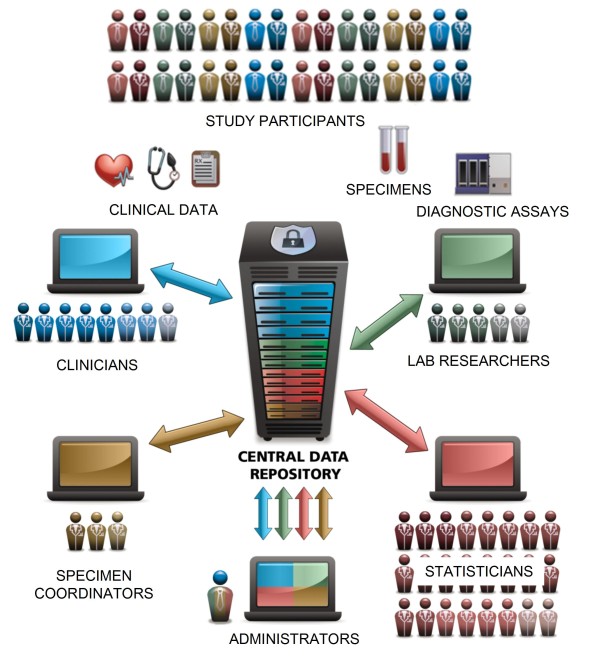
**The Atlas data repository**. Atlas facilitates collaboration among scientists, clinics and labs distributed across the globe by acting as a centralized, integrated, secure data repository. It currently serves the following members of the Global HIV Vaccine Enterprise: the Center for HIV Vaccine Immunology (CHAVI), the Collaboration for AIDS Vaccine Discovery (CAVD), the HIV Vaccine Trials Network (HVTN), the Microbicide Trials Network (MTN), the Vaccine Immunology Statistical Center (VISC) and the HIV Prevention Trials Network (HPTN).

The SCHARP team found existing software alternatives to be insufficient, so team members collaborated with the LabKey Software team to enhance the LabKey Server platform and to establish Atlas. Atlas is an installation of LabKey Server customized with interfaces specific to Enterprise studies. Atlas does not aim to meet all needs of all researchers within the Enterprise; instead, its core mission is to tie together many different lab systems and data sources, as shown in Figure 1.

### Significance of Latest Enhancements

Recent improvements to LabKey Server have emphasized scenarios that support Atlas's role as an information hub. These enhancements are significant in providing:

(1) **Specimen requests and tracking**. Users can track specimen records, execute web-based requests for specimens and integrate specimen information with clinical data and complex experimental results. No other platform known to us supports all of these scenarios.

(2) **Management of experimental data types that are invented or modified as projects evolve**. LabKey Server's graphical assay design tools are novel in the way they allow scientists to quickly describe and manage arbitrary assay data types, plus extend built-in assay types. Users can graphically associate a broad range of scientifically-relevant properties (*e.g*., regular expression validators and standardized out-of-range markers) with each column of assay data and metadata. These properties can facilitate quality control, visualization and analysis.

(3) **Integration, analysis and visualization of diverse data sources**. The platform's tools for creating custom, integrated views of data are exceptional in spanning not just built-in data types and sources, but also user-extended data types. Furthermore, LabKey Server is the only open source system known to us that allows users to integrate clinical data, specimen records and complex experimental results by leveraging: (i) basic relationships between study entities (*e.g*., participants, cohorts, visits and specimens) (ii) SQL-based queries and (iii) graphical view-building tools. The system is also noteworthy for providing dynamic access to external data sources.

(4) **Extensibility**. It is unusual for a scientific data management system to provide backwards-compatible, well-documented client libraries that enable developers to both interact with stored data and to construct custom interfaces. It is also unusual for a system to provide such rich client libraries that developers do not need to become well-versed in the system's object model to quickly develop rich content. Lastly, LabKey Server's built-in, web-based interface for writing and deploying custom R scripts is also exceptional among data integration platforms.

## Implementation

### Architecture

LabKey Server is a web application implemented in Java that runs on the Apache Tomcat web server and stores its data in a relational database engine, either PostgreSQL or Microsoft SQL Server. An earlier version of the platform was called CPAS (Computational Proteomics Analysis System) [49]; the current version includes all of the features of CPAS. LabKey Server is supported on computers running Microsoft Windows and most Unix variants, including Linux, Macintosh OSX and Solaris. Production installations can be upgraded in place with minimal down time. Some installations are run as software-as-a-service (SaaS), which moves server management out of the lab.

**Figure 2:**
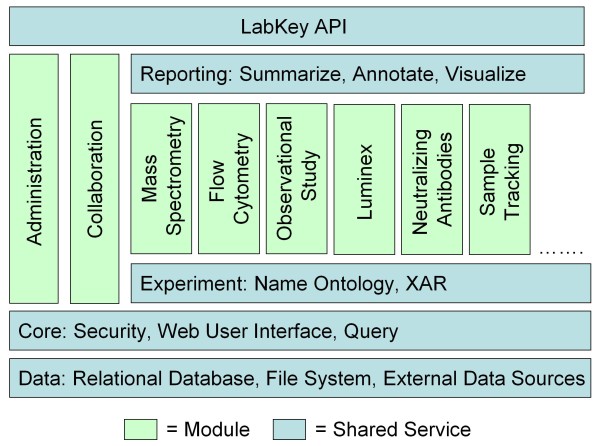
**LabKey Server's modular architecture**. LabKey Server consists of core services and specialized modules. Modules are autonomous, so they can be added or upgraded independently to add new functionality.

Figure 2 shows that the system consists of core services, including data storage, file management and security, together with specialized modules. LabKey Server modules support specific scientific scenarios by encapsulating application logic, user interfaces and data. Data can be shared and integrated across modules. Modules can be added, upgraded, distributed or removed independently, allowing the addition of new analytic capabilities, support for new types of data or other features. On an individual basis, they can be kept private within an institution or contributed to the LabKey Server open source project.

Datasets that reside in external repositories can be made directly accessible through a LabKey Server. Access to such datasets is dynamic, meaning that any modifications to such datasets within an external repository are immediately viewable on the associated LabKey Server. Dynamic access can be configured for PostgreSQL, MySQL or Microsoft SQL Server databases, or for other data sources such as SAS[50]. In general, users can work with data from external sources just like any other type of data on a LabKey Server. Authorized users can view shared datasets using LabKey Server's familiar, accessible grid user interface. Users can customize their views with filters, sorts and column lists. They can use the datasets in custom queries and reports, or export the data to Excel, web query, or simple text formats. For data sources other than SAS, changes can be made to datasets in the external repository using the LabKey interface. While datasets from any one data source can be joined to each other, datasets from different data sources cannot yet be joined directly.

### Basic Platform Services

LabKey Server's role-based security model allows tight control of access to sensitive data while permitting broad sharing of content when this information is ready for wider release[49]. Users can be assigned specific permissions outside of their groups and roles, allowing fine-grained control of access. Workspaces on a LabKey Server are arranged hierarchically and permissions can be inherited by children. Permissions are enforced no matter how information is accessed, including full-text search, data export and the LabKey API (Application Programming Interface). Updates to administrative settings and scientific data on a LabKey Server are logged, enhancing security and enabling auditing.

Authentication of users occurs either through LabKey Server's core authentication system or through external authentication systems. A LabKey Server installation may optionally connect to an LDAP (Lightweight Directory Access Protocol) server to automatically authenticate users within an organization. LabKey Server also supports Single Sign-On (SSO) through OpenSSO[51], allowing authentication of users from a partner web site.

The system provides full-text search for most types of data and documents, plus "science-aware" search for relevant concepts, particularly participant identifiers and study properties. A server can also be configured to display search results from external web sites. LabKey Server also provides a variety of web-based collaboration tools, including file management, wikis, message boards and issue trackers[49].

Automated exception reports are generated by LabKey Server installations and reported back to LabKey Software. By monitoring exception reports, the LabKey team can quickly fix issues and publish patches without the need for users to report these problems.

### Customizable Data Types

A key challenge of scientific data integration is the diversity and the rapidly changing nature of the data types that must be integrated. LabKey Server meets this challenge by combining the flexibility and rich metadata capabilities of RDF (the semantic web's Resource Description Framework) [52] with the regular structure and familiar query mechanisms of a SQL (Structured Query Language) database.

The semantic web defines a network of interconnected resources, each of which can be uniquely identified by a Uniform Resource Identifier (URI). These resources are described by a set of properties and property values. Because the properties and property values are themselves resources, rich data and metadata can be assigned to every resource. Following the semantic web model, data items stored in a LabKey Server can be addressed with a URI in the form of a Life Sciences Identifier (LSID)[53]. Furthermore, they can be associated with an extensible set of properties known within a LabKey Server as fields.

LabKey Server provides a set of basic, predefined data types that can be extended with custom, administrator-defined fields. These data types include lists, assays, study datasets, and specimens. Fields may include standard SQL data types, such as string and numeric types, but may also use semantically richer property types designed for scientific research, such as participant identifiers. Fields can be associated with other scientifically interesting properties, such as out-of-range values, custom indicators for missing values, regular expression validators and custom URL templates for generating hyperlinks to external or internal resources. Fields can also be annotated to indicate that they represent concepts described in curated ontologies, such as those provided in UMLS (Unified Medical Language System)[54]. LabKey Server also allows administrators to define lookup properties that behave as foreign keys and allow automatic joining of related data.

### Query Service

**Figure 3:**
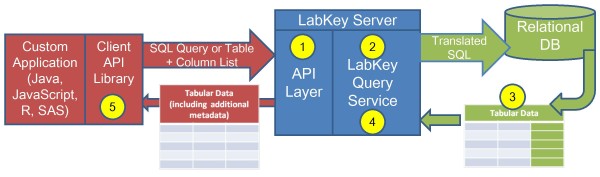
**LabKey Server query service**. The query service can be called on by LabKey Server's APIs or web-based interface. In either case, the service receives a request for one of the following: • A table and a column list. A column list can include columns from the requested table or columns from related tables. • A SQL query based on pseudo-tables known to the query service. For an API call, the following sequence of events occurs: 1. When the request is received by the server's API layer, the layer checks folder security and translates the request into calls to the query service. 2. The query service then uses schema information describing physical tables and pseudo-tables to translate the input query into a SQL query of physical tables. The query is formulated in the dialect understood by the underlying relational database. Schema information is supplied by other LabKey modules. 3. The database returns a tabular result. 4. The tabular result is annotated with additional information about the columns (***e.g***. user-friendly label, description and formatting hints). 5. The appropriate LabKey client library converts this standard data/metadata into a form easily understood by the client language. For example, an R dataset would be returned as the result of a call by an R client API.

All LabKey Server data types benefit from a core query service that allows users to browse, sort and filter tabular data. This service is diagrammed in Figure 3. It supports a graphical interface that allows users to create customized data views and save these views for reuse and sharing with other users. LabKey Server's built-in tools for creating R views, building crosstab views and drawing simple charts all leverage the query service. The query service also allows developers to write full SQL queries that can be executed by other users. Finally, the service provides the ability to export tabular data in a variety of formats for analysis with external tools.

To maintain security, the query service interprets these queries and executes them over a virtual database schema that reflects the permissions of the currently logged in user. For example, the system can perform cohort blinding by prohibiting particular users from viewing data *columns *that would reveal the cohorts of participants. Similarly, if a clinician holds permissions sufficient only for viewing data for locally enrolled participants, the clinician can only access views that are customized to hide data *rows *for all other participants.

## Results

Recent enhancements to the LabKey Server platform enable four core scenarios, all of which have contributed to the success of Atlas. Full documentation and tutorials for LabKey Server are available at http://www.labkey.org.

### Scenario 1: Specimen Requisition and Tracking

LabKey Server's specimen management system provides more than a centralized repository of specimen information. It also supplies secure, web-based tools for requesting, approving and tracking specimen transfers between clinics, repositories and labs. Centralized specimen information can be annotated and integrated with clinical, assay or other data for study participants or animal subjects to allow comprehensive analyses.

**Figure 4:**
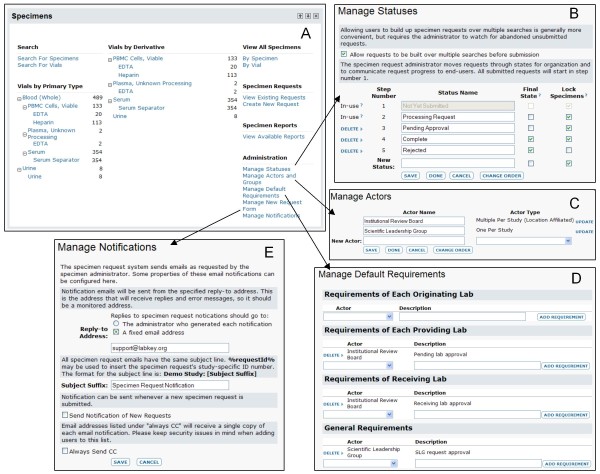
**Typical setup steps for specimen tracking and requests**. 1. An administrator sets up a folder to display the "Specimens" web part and configures permissions for those who will be interacting with specimens. The administrator then imports an initial archive of specimen records and information about repositories and sites. A populated "Specimens" web part is shown in **A**. 2. A specimen data manager configures the specimen request process, including configuring request steps **(B)**, identifying actors in the request process **(C)**, configuring requirements for request approval, and setting up notification procedures **(D)**.

**Figure 5:**
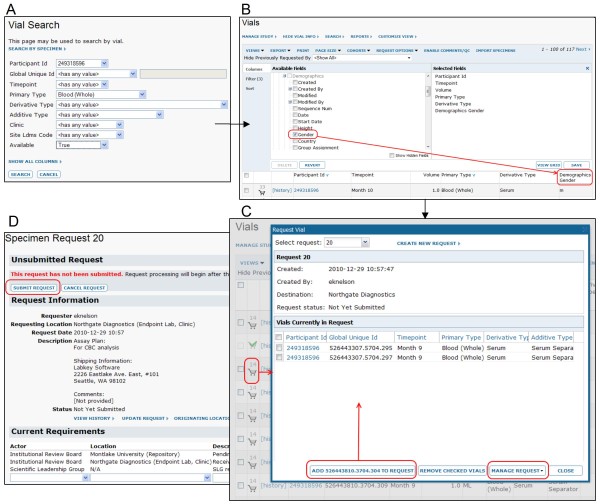
**Typical steps for vial search and request**. 1. A scientist logs on the system from a participating lab, then searches for vials or specimens of interest, as shown in **A**. 2. To further narrow down the possible vials of interest, the scientist builds a custom view of the subset of vials she has identified through search. This custom view integrates information from diverse sources using shared identifiers. In the example shown in **B**, shared participant and visit identifiers are used to join in data from a related dataset. In this case, the gender of the participant who provided the sample (as defined in a separate, demographic dataset) is drawn into the vial data view. 3. The scientist then creates a new specimen request and uses the "specimen shopping cart" to add desired vials to this request, as shown in **C**. 4. When finished, the scientist submits the finished request, as shown in **D**. 5. Designated reviewers are notified of the requests and approve them. 6. Specimen repository workers are notified of the approved requests, fill the requests and update the web-based interface. Vial(s) that have been used are no longer requestable. 7. After receiving a specimen vial, the scientist performs an assay on the specimen. Results from this assay may be marked with the vial identifier or participant/visit identifiers so that they can be associated with specimen information on the LabKey Server. The process for associating assay results with specimens is shown later in Figure 7.

Figure 4 walks through typical steps for importing specimen information and configuring the request process. Figure 5 reviews a simple usage scenario, including searching for available specimen vials, joining specimen data to related data and requesting specimens using the specimen shopping cart.

LabKey Server also includes built-in tools for building specimen summary reports that allow data managers to leverage the centralized availability of information about specimens. Reports can be parameterized by the type of specimen, date of collection, availability of vials, source participant in the study, cohort of the participant, current location and other measures.

This specimen management system is complementary to pre-existing, site-specific tools. Most labs already have LIMS, such as LabWare[55] or the Frontier Science Laboratory Data Management System (LDMS)[56], for specimen management. These LIMS are typically set up with freezer layouts, technician identities, mailing addresses, workflow info, and the like. The LabKey specimen management system does not aim to replace lab-specific LIMS; instead, it serves to connect them. Cross-site specimen management is typically a missing piece for LIMS that handle specimens, so LabKey helps consortia to "glue together" their LIMS through cross-site tracking of specimens and specimen requests.

Members of the Enterprise use Atlas heavily for both specimen request management and integration of specimen data with other types of data. The system records approximately 860,000 specimen vials and 1,300,000 vial transfers. Additional usage statistics are covered in the "Atlas Usage" section of this document.

### Scenario 2: Management of Experimental Data

Typically, labs manage new types of experimental data in spreadsheets, but this can quickly become unsustainable as results proliferate. LabKey Server provides graphical tools for describing, importing and analyzing assay data that would otherwise reside in a multiplicity of spreadsheets. These tools make it easier to bring data straight from the bench into a common system, minimizing the cost of centralizing data, preserving data provenance information, enhancing standardization of data collection and enabling data integration. Assays can also be customized through the LabKey client libraries to include specialized analysis capabilities.

Lab data managers define custom assay "designs" to formally describe experimental results, then import many sets of experimental results to a LabKey Server using the formats specified in the designs. The structure of an assay may include the number of input samples; the type and format of experimental result files; and the definition of summaries or visualizations appropriate for sharing.

Defining experimental properties in the form of an assay design helps to ensure that appropriate data points are collected for each experimental run or set of runs loaded into the server. For any manual data entry steps, LabKey automatically generates the appropriate data entry pages based on the assay design. The design determines which data entry elements are required and which are optional. A lab technician can also use the assay design to set appropriate default values for data items or provide pick-lists of standard values. This reduces the burden of data entry and the incidence of errors.

Customized assay designs can be based on a general template, or on specialized assay types that are added to the LabKey platform as modules. Specialized assay types currently include: neutralizing antibody (NAb); enzyme-linked immunosorbent spot (ELISpot); microarray; Luminex; cell recovery and viability; complete blood count; particle size, high performance liquid chromatography; and enzyme-linked immunosorbent assays (ELISA). Some of these have been developed to match the structured output of tools used by existing platform users, so they can be instrument-centric. Just like LabKey Server's proteomics and flow cytometry tools [49,57], all assay types are backed by a common experimental design architecture that defines notions of experiments, runs, batches (groups of runs), protocols, inputs, outputs and materials (specimens, samples or tissues)[49].

Assay run creation and deletion are audited and run data cannot be modified after runs are imported. Annotations can be added to assay runs through the user interface or programmatically through quality control scripts. LabKey Server's assay infrastructure can support GCLP (Good Clinical Lab Practices)[58] and the establishment of repeatable, reliable, auditable and comparable lab procedures.

**Figure 6:**
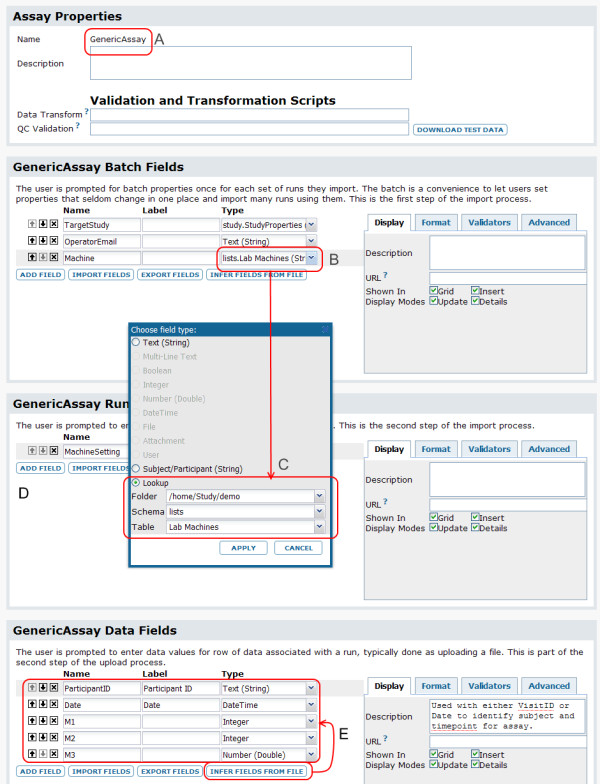
**Typical steps for designing an assay**. 1. An administrator creates an assay-type folder to act as a staging area for assay data before the data has undergone quality control. 2. A scientist creates a new assay design to match the contents of experimental results stored in spreadsheets. In this example, the assay design is based on a generalized assay template, but other assay types could be used. The assay design is named "GenericAssay," as marked by **A**. 3. The scientist then adds a set of batch fields that must be filled out when each batch of runs is imported. User-defined batch fields are backed by the same set of customizable properties as system-defined fields. This image shows label, description, type, lookup and custom URL properties for several fields. Other properties include conditional formatting, default values and regular expression validators. Here, the "Machine" batch field (**B**) is configured (in popup **C) **as a lookup to a simple list ("Lab Machines"). At the time of data import, the user will be presented with a defined list of options for populating the "Machine" field that are drawn from the primary keys of the "Machines" list. 4. Next, the scientist defines the run fields (**D**) that must be collected for each run. In this example, only one field ("MachineSetting") is defined. 5. Finally, to save time, the scientist infers the assay's data fields (**E**) from a representative spreadsheet file. These fields could also have been designed manually, in the same manner as the batch and run fields.

**Figure 7:**
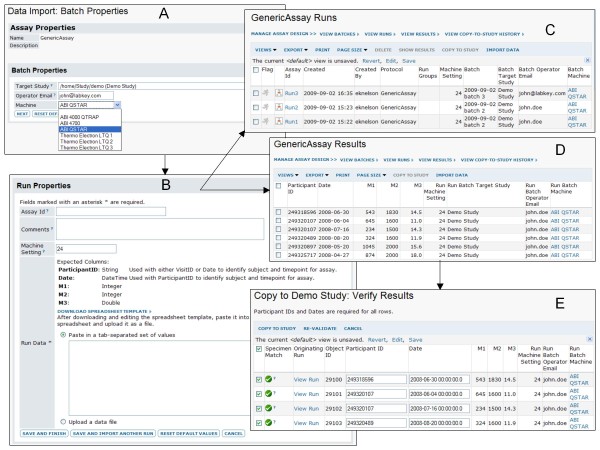
**Typical steps for populating an assay**. 1. The user selects the assay design that matches experimental data and chooses to sequentially import run data. This example uses the assay design from Figure 6. 2. For each batch of data, the user is prompted for batch properties (**A**). Here, the "Machine" options are provided as a defined vocabulary to reduce errors and variability in data entry. 3. For each run, the user is prompted for run properties and run data (**B**), as described in the assay design. A template of expected columns can be exported to help with matching data formats. 4. The user can import several runs sequentially using the same batch properties. **C **shows a summary view of the three runs that have been imported to this assay design using the same batch properties. 5. **D** shows an example of results imported as a single run for this assay. Note that the "Machine" column is defined as a lookup to another table, so each of its entries is hyperlinked to details for the appropriate machine, as provided by the "Lab Machines" list. 6. After assay data has been reviewed for quality control, it can be moved into a study folder for sharing with collaborators and integration with other types of study data. During the import process, the participant/visit identifiers for each row of assay data are matched (**E**) to identifiers for specimens in the target study. This allows viewers of the assay data within the study to quickly navigate to data for associated specimens.

Figure 6 shows typical steps for designing an assay, while Figure 7 shows typical steps for populating the same assay design.

**Figure 8:**
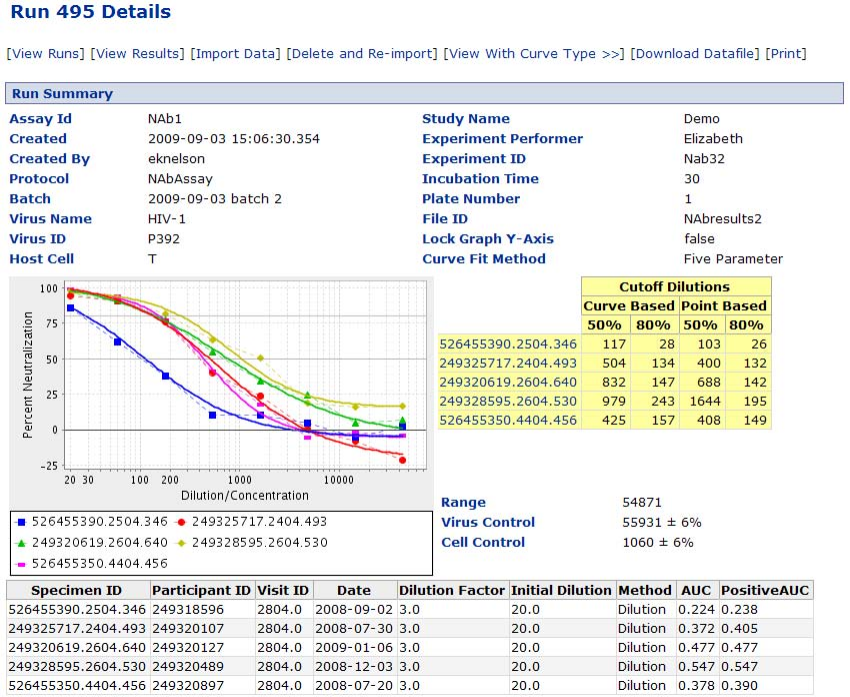
**Detailed NAb assay results view**. The "details" view for a NAb assay visualizes measures of neutralization success and provides several calculations for areas under the neutralization curve.

LabKey Server's neutralizing antibody assay provides an example of how the system's assay tools can encourage process standardization across labs and catalyze contribution of data to a central repository for integrative analyses. The NAb assay tool included in the LabKey platform was developed to formalize data management for the TZM-bl NAb assay [59]. Replacing a spreadsheet macro, it simplifies data processing by providing an automated system for uploading, transforming and analyzing data and displaying results (shown in Figure 8) through a web-based interface. Data from the plate reader and metadata describing the experiment are imported to the server, where calculations are done automatically and results can be visualized and shared.

As part of Atlas, the NAb tool has been used successfully by 14 labs across 4 organizations within the Enterprise (CHAVI, VISC, HVTN and the U.S. Military HIV Research Program). As of May 2010, these labs have used the NAb assay tool to upload and store over 25,000 NAb assay runs. The labs use the tool not just because it enables data transfer, but because it provides immediate value. The tool provides technicians with graphical feedback that indicates whether results fall within expected bounds, and thus whether the assay has been performed correctly. The use of the NAb tool facilitates standardization, organization, auditing and integration with other types of repository data, such as specimens.

### Scenario 3: Data Integration

Users of LabKey Server can draw together information stored in multiple tables using built-in summary views, a graphical cross-source view designer and custom SQL queries. Datasets are typically connected through shared identifiers for subjects (*e.g*., participant, animal or subject identifiers), samples (*e.g*., specimen identifiers) and/or time points of data collection (*e.g*., participant "visits" to clinics). However, tables do not need be related through these types of identifiers to be joined into common views; they may also be joined through administrator-defined lookup fields. Joined, integrated views can be used as the basis for complex analyses and visualizations.

**Figure 9:**
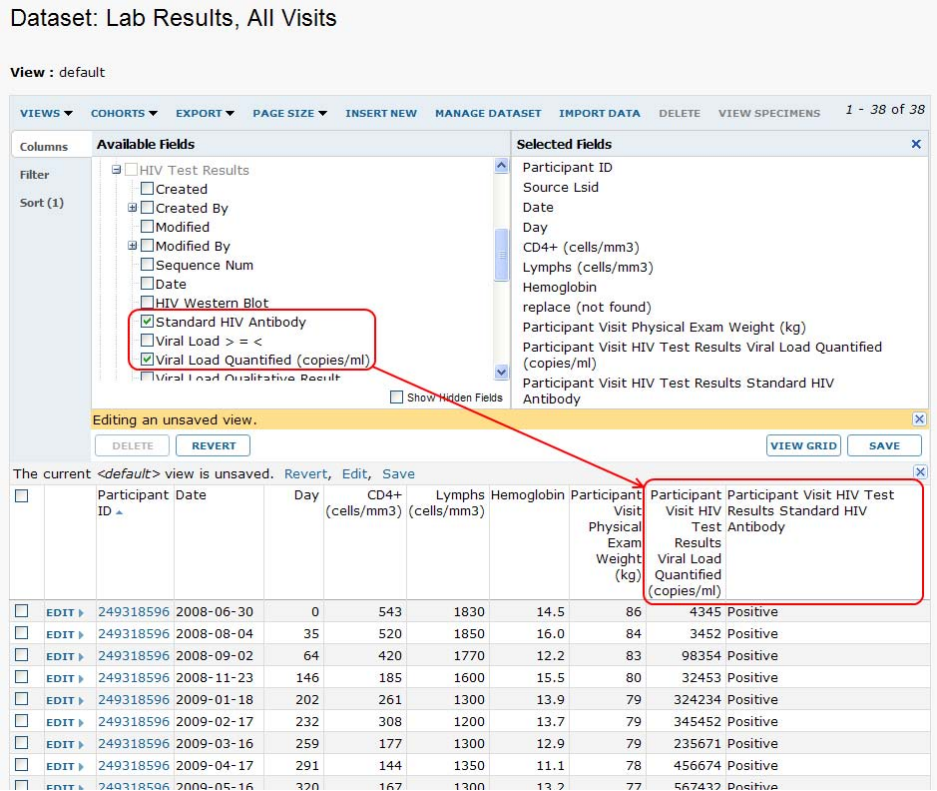
**Example of joining datasets based on built-in relationships**. Results from related data sources can be joined into a common grid view using built-in relationships between study entities. Here, the grid view customization tool is used to pull in columns from data sources that include the same participant/visit pairs. The results of an assay ("Lab Results") are joined with the columns from another assay ("HIV Test Results") and the results of a physical exam ("Physical Exam"). The joined custom view can be saved privately, made visible to collaborators or exported in various formats, including Excel.

**Figure 10:**
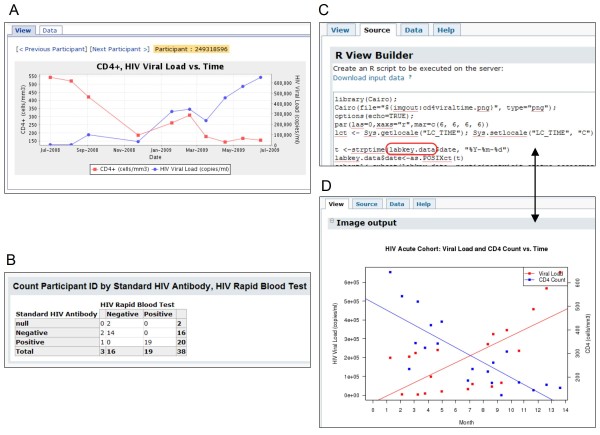
**Examples of analyzing and visualizing a joined custom view**. Here, the joined custom view created in Figure 9 is used to construct a participant chart (A), crosstab view (B) and R chart (C and D) through the "Views" menu available above the joined grid. A. The participant-specific chart view marked by A displays the same type of information for each study participant, in sequence. Users can toggle between the displayed chart and its associated dataset using the "View" and "Data" tabs. This particular chart displays the progression of HIV viral loads and CD4+ counts over time for each study participant. B. A crosstab view like the one shown in B could be used to verify that different tests for determining HIV status produce consistent results. C. The R script displayed in the "Source" tab of the R script editor (C) uses data from the joined custom view to compare how CD4+ counts and HIV viral loads change over time. The scripting environment makes the source dataset available as the labkey.data data frame, as circled in red. D. The results of the script are displayed by selecting the "View" tab of the R script builder, as shown in D. Selecting the "Data" tab would display the source dataset (not shown). R views, just like other views, can be saved privately or made visible to collaborators with sufficient permissions to view the source data. Within LabKey Server's R environment, users can also invoke stored scripts and leverage advanced analysis or visualization packages, such as those provided by Bioconductor[80].

**Figure 11:**
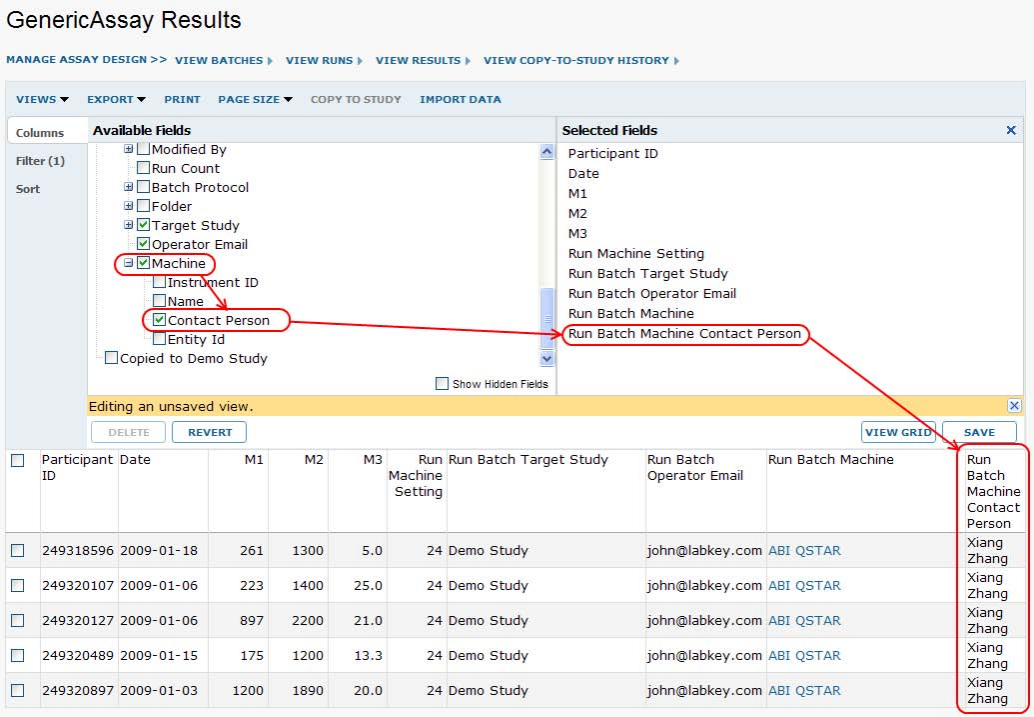
**Example of joining data sources through a user-defined lookup relationship**. The "Lab Machines" list, a simple table, was used in Figure 6 to provide a defined vocabulary for populating the "Machine" batch field of the "GenericAssay." The "Machine" batch field was defined as a lookup to the "Lab Machines" list. The existence of this lookup relationship allows us to join machine information into grid views of assay run data. Here, as circled in red, the relevant machine's "Contact Person" is joined into a grid view of assay results.

**Figure 12:**
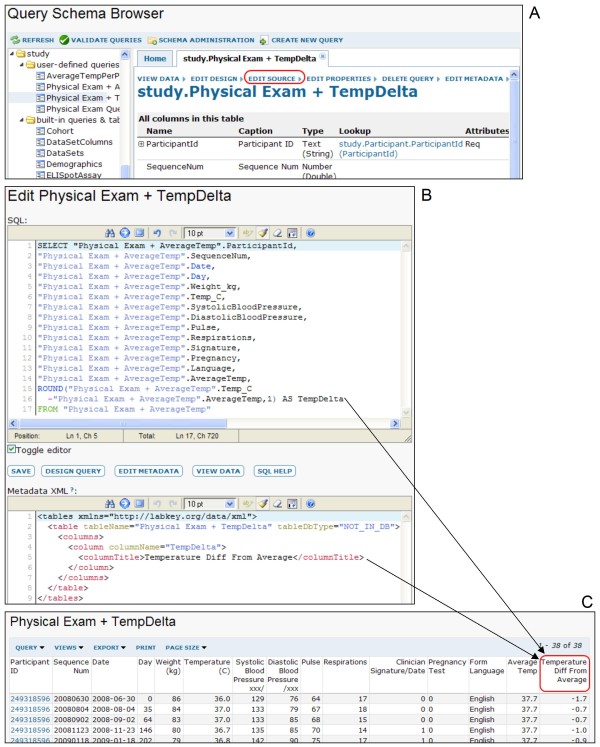
**Example of creating a custom SQL view**. This figure demonstrates how a custom SQL view can add a calculated column to a joined view and label the column using custom metadata. Part A of this figure shows LabKey Server's schema browser, which allows a developer to view, add or edit custom queries. Part B shows how the SQL source editor has been used to add a calculated column to a table as part of a custom query. It also shows how the table metadata editor has been used to edit the column's properties and add a custom title. The grid view produced by this custom query is shown in C.

Figures 9 and 10 show how LabKey Server's graphical tools and R can be used to join, analyze and visualize data from multiple source tables based on participant/visit identifiers. Figure 11 shows how the system's custom view designer can construct a joined view by means of a user-defined lookup relationship between two tables. Figure 12 shows how LabKey Server's SQL editor enables the construction of more sophisticated queries, including the inclusion of calculated columns and custom metadata. All of these figures use made-up data.

**Figure 13:**
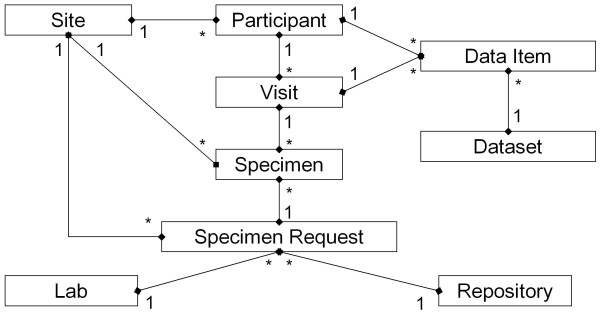
**Simplified study schema**. This simplified schema outlines the relationships between entities in a study. Data tables in a study can be integrated when they contain shared quantities, such as participants, visits or specimens.

On a LabKey Server, a folder-based "study" serves as the primary integration point for connecting heterogeneous data types collected as part of an observational study. A study defines built-in relationships between study data entities (shown in Figure 13) and provides built-in tools for summarizing and visualizing related data.

**Figure 14:**
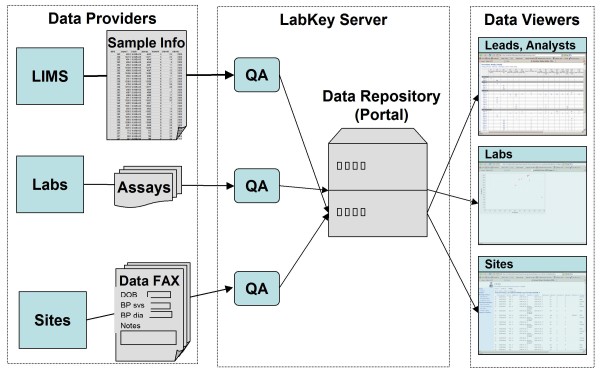
**LabKey Server data flows**. Data flows into a LabKey Server from diverse sources, undergoes quality control and becomes available to a range of data customers through a web-based portal.

Figure 14 shows how data flows into a study in many forms (*e.g*., Excel, text and DataFax case report forms) from many sources (*e.g*., labs, clinics and repositories), where it can be combined and consumed in different ways by collaborators (*e.g*., labs, principal investigators and statisticians). For example, labs might use aggregated data to identify issues with quality control methods, while statisticians might apply novel transformations in R, while principal investigators might monitor overall progress of cohorts through summary views. Studies also provide mechanisms for formalizing data approval prior to sharing and integration; adding "quality control" annotations at the level of datasets or data points; exercising fine-grained control over dataset security; grouping subjects by cohort; enforcing cohort blinding; summarizing data by participant or other measures; and exporting/importing/reloading entire studies for efficient backup, staging or transfer to new locations.

**Figure 15:**
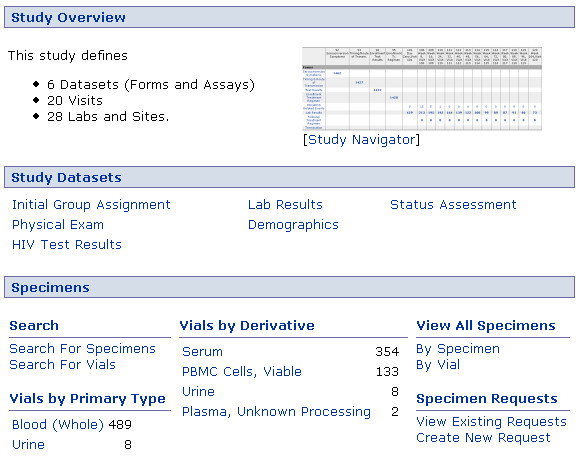
**Study portal page**. A study portal provides a jumping-off point for investigating patterns in shared datasets. The interface can be customized to list the different types of data or views available in the study and to display custom data tables or visualizations. The "Study Navigator" shown in this figure is a data summary tool that rolls up information from many study datasets based on participant identifiers.

**Figure 16:**
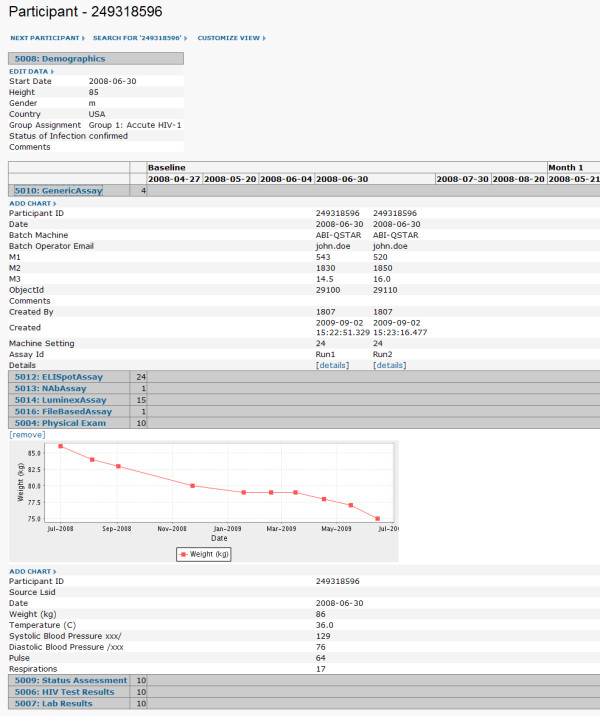
**Participant-specific data summaries**. A data overview is provided for each participant in a study. It displays all datasets in the study associated with a particular participant across all visit dates. Participant-specific charts can be displayed for each dataset, as shown for the "Physical Exam" dataset. A user can navigate through equivalent views for other individuals using "Next Participant" link and "Previous Participant" links.

Figure 15 shows a typical study portal page that lists the datasets and specimens associated with the study. Figure 16 shows the system's built-in interface for viewing all study datasets available for a particular individual across all visit dates.

Atlas exemplifies how a LabKey Server can draw upon both LabKey-based data and data from external systems to support observational studies. Atlas's flexibility in interfacing with external databases allows it to be different things to different types of data -- a database of record, an integration point, or both. Atlas interacts with several SAS and PostgreSQL databases in real time. It also imports data exported from other data sources, particularly a database of DataFax case report forms, and deposits data into relevant Atlas-based studies. For certain types of data (*e.g*., specimen requests), Atlas is the database of record. For others (*e.g*., specimen cell counts), Atlas is simply the integration point for diverse, specialized databases of record.

### Scenario 4: Extensibility

LabKey Server's deep support for customization and rapid application development frees labs to independently adapt their servers, interfaces and analyses to lab-specific needs. Client libraries in a range of languages, plus a user interface for R scripting, allow investigators to use familiar tools to build custom applications, interfaces, assays, reports and analyses. Developers can add larger features by encapsulating them in modules, create individual data views in R or simply add API-enhanced content to wikis or HTML pages in the file system.

LabKey Server's client libraries are backwards-compatible, well-documented and designed to be accessible to developers with varied skill sets, from Java programmers to R scripters. The client libraries provide programmatic access to LabKey Server modules and services (shown in Figure 2) through familiar languages such as JavaScript, Java, R, SAS and Perl. Developers who prefer other languages, such as PHP, can interact with a LabKey Server through JSON over HTTP. Familiarity with LabKey Server's object model is not necessary to quickly produce useful applications.

All client libraries allow users with appropriate permissions to select, insert, update and delete records on a LabKey Server. The JavaScript library also includes APIs for building user interfaces and executing actions commonly performed through the user interface. These include adding web parts, adding users or groups, checking permissions, executing SQL queries, populating datasets, sorting and filtering grid views, requesting specimens, adding folders, building charts, navigating, and building interactive grid views, among many other actions. For example, the entire process of populating an assay (as shown in Figure 7) can be accomplished through the JavaScript client library.

LabKey Server provides multiple of methods for working with R. Users can employ the R client library to load live data from a LabKey Server into an external R environment for analysis, provided the user has permissions to read the data. The R library also supports querying for available data, then inserting, updating, and deleting data records, given sufficient user permissions. In addition to the R client library, the system provides an interactive R scripting interface that allows users with appropriate permissions to author scripts, view script results and see source data, as shown in Figure 10. Lastly, R scripts can be included as files in custom modules to define custom views for custom queries.

The SAS client library provides very similar functionality to the R client library, enabling interaction with LabKey Server data from SAS.

At present, the JavaScript client library is LabKey Server's most fully featured. While the actions available through its APIs are broad and deep, they are not yet completely comprehensive. For example, it is possible to define a new assay type (e.g., a new plate-based assay like the NAb assay) using the client libraries, or to populate an existing assay design (as shown in Figure 7), but it is not yet possible to create a new assay design based on an existing assay type, as can be done through the user interface (shown in Figure 6). Due to high interest among user-funders, LabKey Server's client libraries are expanding quickly.

SCHARP developers have leveraged LabKey Server's client libraries extensively to quickly meet the needs of evolving studies. For example, a custom application built on Atlas was used for adjudicating the results of the Thai Phase III HIV vaccine study, also known as RV144[60]. This trial provided the first modest demonstration of a positive effect for an HIV vaccine. An independent, globally distributed committee judged participant HIV status during this trial by evaluating Western Blot images and other data through the Atlas interface. Using Atlas, committee members travelling between research sites could log on to a web-based interface from locations across the world, view images and enter findings. The process was formerly conducted through postal mail. A single developer created the custom RV144 reporting tool in JavaScript in a matter of weeks. The tool is available only on Atlas.

HTVN has built custom data summaries (available only on Atlas) that allow central labs to view the cumulative success of individual technicians in processing and preserving blood cells, as measured through cell viability tests. Study managers can use these summaries to swiftly identify problem areas and improve quality control. The result is a shorter feedback loop between central labs and remote labs. The summaries also provide transparency to project funders and digital historical records that take the place of paper-based tracking.

**Figure 17:**
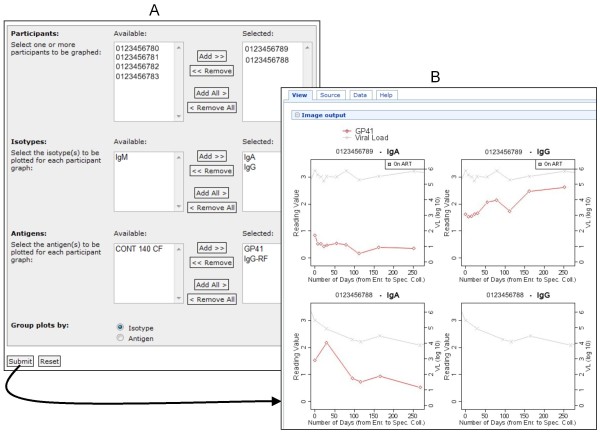
**A custom, interactive form for designing an R view**. Part **A **of this figure shows an example of an interactive R chart designer. This particular interface (available only on Atlas) was created using the LabKey JavaScript client libraries. It allows users to create custom views of antibody binding data. Users employ a graphical form to select participants of interest, isotypes, antibodies and groupings for plots. The form sends user selections to a parameterized R script that runs on a joined view of relevant datasets. Part **B **of this figure shows the R view produced by this form. It shows binding antibody graphs by isotype (interferon alpha (IgA) and interferon gamma (IgB)) for two study participants. The graphs plot glycoprotein 41 reading values (GP41) and viral loads for each participant and each isotype against the number of days that have passed between enrolment of the participant in the study and the collection of the tested specimen. Participant identifiers in this figure have been modified to hide study participant identities.

**Figure 18:**
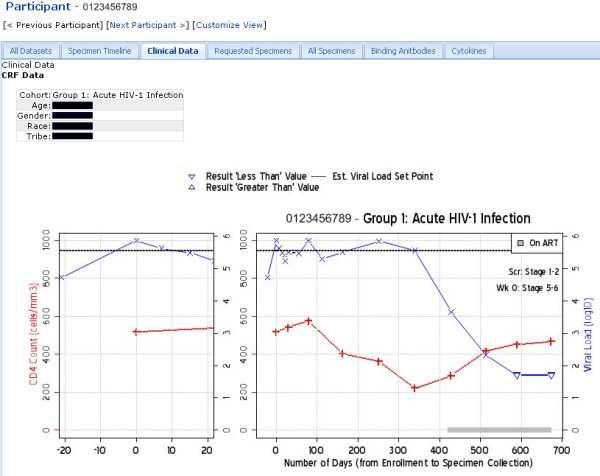
**Custom, per-participant views of data**. Participant-specific summary views show all data available for a particular participant, typically along with visualizations. These views can be customized using the graphical interface or through custom scripts. This figure shows a custom view built on Atlas using LabKey Server's R interface and JavaScript client library. It is available only on Atlas. Clinical data for the selected participant are displayed alongside a graph that plots viral loads and CD4+ counts against the number of days from enrolment in the study to specimen collection. Users can toggle between different types of data (***e.g*.**, "Requested Specimens," "Binding Antibodies" or "Cytokines") using tabs. Just as in the per-participant views shown in Figure 10A and Figure 16, users can employ the "Previous Participant" and "Next Participant" links to progress to the same data summary for other participants in the study. The participant identifier in this image is not real and the physical information for the participant has been obscured.

**Figure 19:**
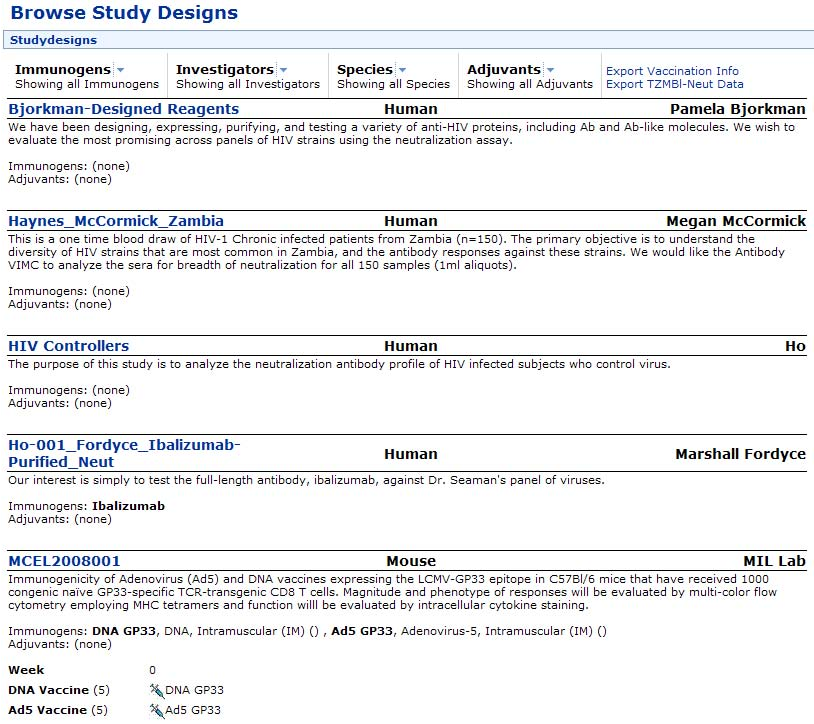
**Custom study summaries defined in an Atlas module**. LabKey Server modules can be used to encapsulate scientifically interesting applications. Atlas's vaccine study module defines several interfaces that generate interactive summaries of vaccine studies, including the interface displayed in this figure. This module is tailored to Atlas-specific data, so it is not part of the open source project. Generated summaries like the one shown in this figure make it easier for scientists to swiftly, publicly share study information and protocols after publishing associated research papers. The Atlas web portal respects user permissions, so study summaries display different amounts of information to different tiers of users. Atlas login is not required to view public study data, so you can explore the interfaces and data summaries defined in the vaccine study module without acquiring Atlas credentials: https://atlas.scharp.org/cpas/viscstudies/VISC/Completed%20CAVD%20Studies/studydesigns.view.

Figure 17 shows how Atlas developers have used LabKey Server's client libraries to build custom, graphical interfaces that enable users who are not skilled in R scripting to generate custom R views. It shows one such interface, plus an example of the type of R view it produces. Figure 18 shows customized, participant-specific views built by Atlas developers using LabKey Server's R interface and JavaScript client library. Figure 19 shows an example of a custom interface defined in a module developed specifically for Atlas. This module defines a custom reporting tool that provides interactive summaries of vaccine studies. It allows users to selectively view information of interest and provides interfaces that roll up summary data for each study. All of interfaces shown in Figures 17, 18 and 19 are available only on Atlas because they are specific to the data Atlas contains.

### LabKey Server Adoption

The ability of LabKey Server to meet core scientific and data management needs in a customizable way is demonstrated by the adoption of the platform by a range of organizations beyond the Enterprise. According to data reported automatically by active LabKey Servers, approximately 40 installations are currently in active use. LabKey Server v10.3, available in November 2010, is the 19^th ^official, public release of the platform.

Adoption of the platform has also meant adaptation; research organizations use LabKey Server for a wide range of purposes. For example, several labs use their installations of LabKey Server to manage the large quantities of data that stream from flow cytometry[57], proteomics[61-64] and/or microarray[61] experiments. Two systems biology labs use LabKey Server to integrate diverse data types at the lab level[61,62]. The National Primate Research Center at the University of Wisconsin, Madison, is customizing a LabKey Server installation to provide an extensible, life-science-aware database for non-human primate electronic health records. These features are being developed as a custom module for LabKey Server that will be available to other researchers. The Primate Center also uses LabKey Server for multiplexed genotyping and next-generation sequencing experiments. A distributed team of cancer researchers based at the Fred Hutchinson Cancer Research Center uses a LabKey Server as a place for "virtual research." Team members consolidate data from existing, online databases onto their LabKey Server and then use R to collaboratively mine this data. Two labs use LabKey Server for post-publication sharing[61,63]. AdaptiveTCR, a T-cell sequencing company, has used LabKey Server to build a customized, proprietary system that allows customers to purchase analyses, submit specimens, view results and interactively visualize data [65]. Insilicos, a company focused on proteomics, uses LabKey Server to support cloud-based, scalable computing[66].

### Atlas Adoption

Atlas's success in achieving adoption across the Enterprise can be gauged by considering usage statistics for the system. The number of accounts (approximately 2,800 accounts across 350 organizations and 36 countries) is notable given the relatively high bar for gaining access to the system, as compared to open-access databases that publish fully anonymized data. Access is restricted due to privacy considerations for clinical studies on a sexually transmitted disease.

Atlas held 2,844 active user accounts in May 2010. The number of individual users likely lies closer to 2,800 because some users (particularly administrators) hold multiple accounts. Approximately 600 additional accounts have been deactivated (as typically happens when an individual leaves a position), so approximately 3,400 total accounts have existed on the system. The first user account on Atlas was created on October 4, 2005.

Approximately 350 distinct organizations are represented among user accounts. Roughly 200 distinct organizations have two or more active Atlas users while approximately 100 distinct organizations have five or more users.

Approximately 36 countries are represented among user accounts. The number of accounts associated with each country suggests the degree of usage in each country. 29 countries were associated with two or more user accounts while 23 countries were associated with five or more accounts.

As of May 2010, Atlas holds 26,684 uploaded assay runs, 2,637 customized data views, 2,116 unique wiki pages and 1,717 message board posts. Also as of May 2010, Atlas has tracked 156,349 specimens (such as blood draws or urine specimens). These have been subdivided into 859,759 vials and transferred during 1,280,407 specimen "events." Events record transfers of all types, including active requests for vials (made through the Atlas interface) and transfers without request (such as automatic transfer of vials from clinic to repository after collection). A total of 801 requests have been entered and processed through Atlas, resulting in the transfer of 19,727 individual vials. Each request typically includes multiple vials.

During April 2010, the Atlas web site welcomed 1,400 unique visitors from 36 countries. The average visit included 13 page views over 11 minutes. Overall, a total of 5,400 site visits produced 70,000 page views during this time period. These statistics are typical of recent months. The number of countries where visits originated was the only measure that notably increased over the past six months (from 27 to 36).

## Discussion

### Lessons Learned

Adopting a shared platform like LabKey Server to accomplish data integration and process standardization can bring network benefits to collaborating organizations. At the same time, achieving adoption of a new platform across a diverse community is not an easy task, even when the community is joined together into a common effort such as the Enterprise. As we have learned first-hand, merely providing *innovative *software features is insufficient-the real challenge is making the software *useful *to scientists.

Other researchers have proposed general principles for developing software for biologists[67-71] or for speeding the broad adoption of innovations[72,73]. However, relatively few[13,74] have explored development guidelines that facilitate adoption of software across biomedical research organizations. We attribute adoption of LabKey Server and Atlas primarily to the use of seven successful development strategies:

(i) **Enable easy extensibility and customization of interfaces, analyzes and visualizations**. Scientific insights often come from nonstandard approaches, so scientists have a natural preference for software that can be customized to the particular needs of their labs. Tools for rapid customization have proven particularly important to both the adoption of Atlas and the dissemination of LabKey Server. For example, SCHARP's development of custom applications on Atlas only took off with the release of LabKey Server's first client API. Before release of this API, development of custom interfaces typically required assistance from LabKey Software engineers. Furthermore, system-level APIs changed often, so custom applications usually broke upon upgrade. Over the first 2.5 years of the life of Atlas (October 2005-May 2008), SCHARP created only 6 custom applications using system-level APIs, for an average of 2.4 per year. In contrast, in the first 18 months after the release of LabKey Server's JavaScript API (May 2008-October 2009), 240 SCHARP-authored applications and tools went live on Atlas, for an average of 160 per year.

(ii) **Add value at the level of the lab bench, not just the overall Enterprise, to entice users to bring data into the system**. Labs are more willing to adopt new data management practises if adoption makes their own work more efficient, standardized and/or reproducible. LabKey Server's TZM-b1 neutralizing antibody tool has met wide adoption because it brings immediate value to front-line labs. It translates key lab workflows into standardized data management practises that enhance efficiency and reproducibility. In contrast, adoption of LabKey Server's Luminex assay tool has been slow because the tool does not provide a clear, direct benefit to labs. It was designed primarily to help labs put their data into a format useful to central data managers. Only 120 Luminex runs were uploaded to Atlas between February 2008 and January 2011 (roughly 40 per year); for comparison, approximately 40,000 NAb runs were uploaded from December 2006 to January 2011 (roughly 9,800 per year).

(iii) **Interoperate easily with existing, external data sources**. Easy interoperability enables data integration without the need to first transform a LabKey Server into the primary or archival repository. For example, LabKey Server is not the database of record for Enterprise specimens. Instead, the Atlas installation of LabKey Server synchronizes with existing LIMS systems. This allows members of the Enterprise to retain existing workflows and avoid transferring legacy data to a new platform. Given the extent of existing systems, the development of Atlas is unlikely to have occurred without interoperability.

(iv) **Practice agile**[75]**, interdisciplinary software development to continually incorporate user feedback**. Close collaboration between data managers, research scientists and independent software engineers ensures continual focus on actual, not theoretical, user needs. The team uses short, four month release cycles and a formal feature review process to tighten the feedback loop. Notably, the features that have received the most user feedback during design and development (*e.g*., the TZM-bl neutralizing antibody assay, specimen management and API libraries) have become the most widely used tools. Features that did not have significant end-user involvement in the design process (*e.g*., the ELISpot assay tool) have experienced the slowest adoption. Notably, not a single ELISpot run has been uploaded to Atlas since the initial release of the tool in May 2008.

(v) **Use a platform-based approach to meet shared needs cost-effectively**. The LabKey team works to identify common, long-term requirements for the platform so that the core system features it builds (*e.g*., the assay designer and tools for file management) meet shared needs using common infrastructure. New, specialized applications can simply leverage these core platform services. This lowers costs, reduces bugs and increases the speed of development. As we have learned, straying from a long-term approach (*e.g*., building a new assay outside of the generalized assay infrastructure) makes certain features (*e.g*., integration of this assay with sample management) cost-prohibitive.

(vi) **Ensure that cross-disciplinary facilitators have bandwidth for adoption**. SCHARP data managers have played a particularly important role in the success of Atlas. They have combined their understanding of research objectives with their knowledge of Atlas capabilities to facilitate the upload and effective use of data, cementing adoption. For new installations of LabKey Server, successful adoption has often been attributable to a primary champion in a lab who has set aside sufficient time to become a skilled user of the platform. Without in-house advocates and experts, adoption has often faltered.

(vii) **Establish a reliable track record for ongoing, professional development, maintenance and support**. Don Listwin of the Canary Foundation has quipped: "[Scientific] open source software has the half life of a graduate student"[76]. Given this type of scepticism and the resources required for adoption, it is particularly important to use development practises that build confidence in the longevity of the system. The LabKey Software team has used decades of experience in building commercial software to establish enterprise-calibre development practices for design, testing, stabilization, deployment and support. Some practises, such as automated exception reporting and public transparency, go beyond those common in the industry. Official builds of the platform are released with regularity, three times per year.

### Limitations of LabKey Server

Most biomedical research organizations have unique and evolving software needs due to their specific suites of pre-existing infrastructure, distinctive organizational processes, and involvement in rapidly changing areas of science. A data integration platform like LabKey Server must therefore be tailored to such an organization's needs before the system becomes useful. The effort to successfully establish an installation of the platform should not be underestimated.

Additional boundaries of the platform stem from its scientific focus. The platform provides little direct support for managing the business side of scientific enterprises. It provides specimen request management and generalized issue tracking, but it does not replace existing tools for such things as ordering reagents, tracking inventory, managing freezer layouts and scheduling work shifts. The platform does not aim to replace mass-market collaboration software, so it does not provide SharePoint-style document co-authoring.

LabKey Server's facilities for tracking disease progression over time focus principally on associating data with individuals (subjects, participants or animals) and time points. These tools are less useful for experimental lab studies that focus on replicates, such as experiments on yeast biochemistry. Studies that require location as a key identifier, such as geographic studies of disease spread, would require support that is not yet built into the platform. The system is not currently designed around the execution of clinical trials; nevertheless, organizations such as HVTN still use it to share and adjudicate results.

At present, the study-based specimen tracking and request system requires that uploaded specimen data conform to a specific format based on the output of a particular LIMS, LDMS[56]. Greater flexibility towards specimen input formats would not be difficult to add. The system already provides other tools for tracking arbitrarily shaped specimen data, but these do not support requests.

LabKey Server provides features (such as role-based security, authentication, audit logging and write-once rules for assay data) that are designed to meet the requirements of FDA Regulation 21 CFR Part 11. However, no installation of the platform has yet undergone full, formal evaluation for compliance. Compliance can only be certified for installations of software, not the software itself.

LabKey provides for data export in multiple formats, but does not yet provide protocols for data transfer to permanent, domain-specific archives, as do ISA and SIMBioMS[11,9].

### Next Steps for LabKey Server in Support of Atlas

A key future focus for Atlas will be the development of new tools for interactive visualization and data exploration. These tools will allow more efficient extraction of information and insight from Atlas. Data exploration features will include interactive graphics, new tabular displays tailored to requests from investigators and tools for quickly performing analysis of variance (ANOVA) calculations and other statistical analyses. The data exploration tools will be combined with improved data submission capabilities, allowing investigators to swiftly and easily combine their own data with data stored on Atlas. In addition, we expect to simplify the application of existing ontologies to data types, allowing richer integration of data across independent datasets. We are also prototyping a distributed HIV dataspace [77] that would provide a catalogue of data stored in a variety of locations.

Additional areas of focus may include the development of new custom assay tools (following the successful model of the TZM-bl neutralizing antibody assay) and the enhancement of full-text search. Integrating deeper knowledge of biomedical concepts into full-text search would better enable searches for scientifically relevant information.

### Additional Next Steps for LabKey Server

Future areas of focus depend on the needs of users who fund further development of the core platform. Enhancements to the LabKey client libraries to aid application development have been a particularly consistent area of focus among user-funders. Support for next-generation sequencing data and integration with Galaxy[78] is currently funded and under development. Adoption of the platform by consortia studying diseases beyond HIV would require certain enhancements, such as new custom data types, but the basic platform has been designed for use by consortia studying any disease.

## Conclusions

Sharing data, analysis tools and infrastructure can accelerate the efforts of large research consortia by enabling new insights and enhancing efficiency. The Atlas installation of LabKey Server demonstrates the utility of the LabKey platform for collaborative research. Like all LabKey Server installations, Atlas supports secure, web-based data sharing and collaboration from the earliest stages of disease research; enables integration of diverse and changing data types based on subject and/or visit identifiers; allows easy customization of interfaces, wizards, analyses, and visualizations; supports programmatic automation and customization; supplies advanced tools for data querying, search and analysis; provides dynamic access to external databases; enables staging of data based on quality control status; and provides specimen request management.

Real-world adoption of Atlas by members of the Enterprise has helped the LabKey Server team refine the features of the base platform to suit the needs of a wide range of researchers. Functionality tailored to be useful to a broad array of scientists has helped to catalyze adoption of the platform beyond the Enterprise. Funding agencies' growing enthusiasm for collaboration among disease researchers[3,79] suggests that the platform will become increasingly useful to a wider circle of researchers focused on other diseases.

LabKey Server's open source license means that other research consortia can freely adapt the base platform to their needs while contributing new features back to the effort and improving the software for all users. The platform's track record of regular, stable releases and ongoing maintenance provide a reassuring complement to its open source availability.

## Methods

This section covers the methods used for measuring Atlas usage. Counts of active Atlas user accounts were made on May 11, 2010, as were estimates of the number of organizations and countries represented by these counts. To estimate the number of organizations using Atlas, we counted the distinct domains used by active user email accounts. This count excluded obvious duplicates (*e.g*., multiple email domains at the National Institute of Health) and obvious commercial, non-organizational email accounts (*e.g*., Gmail, Yahoo and others). The total count may be an overestimate because there could have been further duplication, so it is useful mostly as a benchmark.

To estimate the number of countries where Atlas is used, each user account was associated with a country of origin based on the account's country code top-level domain. If the domain did not include a country code (*e.g*., .com or .org domains), the domain was assigned to the United States category. This practice made the country count a conservative estimate.

Overall counts of assays uploaded to Atlas, customized views, wiki pages and message board posts were made on May 11, 2010 by querying the production server's PostgreSQL database. Counts of specimens were made on May 17, 2010 on the staging server, whose content mirrors the production server with only a slight time lag. NAb, Luminex and ELISpot assay runs were counted on the staging server on January 10, 2011 for the purpose of comparing adoption rates for these assay tools. Counts of SCHARP-authored applications and tools on Atlas were made on the production server in October 2009 and included wiki-authored tools, file-based applications (excluding static content) and full-fledged modules.

Traffic to the Atlas web site was measured through Google Analytics. Tracking began in July 2008. Measurements for the month of April 2010 were compared to measurements in October 2009 to estimate current trends in usage.

## Availability and Requirements

### LabKey Server Open Source and Compiled Binaries

The LabKey Server open source software is freely available for download at http://www.labkey.org under the terms of the Apache License 2.0 [1]. This site also provides documentation, tutorials and demos for users and developers, plus instructions for developers who wish to contribute code to the project through the LabKey Subversion repository.

Compiled binaries for Windows, Unix, Linux or Macintosh installation are available for free through LabKey Software at http://www.labkey.com. A graphical installer is available for computers running Windows XP or later. It includes the LabKey web application; the Apache Tomcat web server, v5.5.29; the Java Runtime Environment, v1.6.0-22; the PostgreSQL database server, v8.3.7; and additional third-party components.

**• Project name: **LabKey Server

**• Project home page: **http://www.labkey.org

**• Operating system(s): **Platform independent

**• Programming languages: **Java, JavaScript, R, SAS, *etc*.

**• Other requirements, as of LabKey v10.3: **Apache Tomcat (5.5.29 or 5.5.31); Java Runtime Environment 6; and either PostgreSQL (8.2, 8.3 or 9.0) or Microsoft SQL Server (2005 or 2008). Check the project site for latest requirements of the most recent release.

**• License: **Apache License 2.0 [1]

### Hardware Requirements

LabKey Server can run on any type of modern computer hardware. Typically, the needs of the database are much greater than the web server, so these may run on different machines. Hardware requirements depend on the load placed on the system. In general, a modern, server-level system running Windows or a Unix-based operating system is sufficient for a modest deployment.

### Access to the Atlas Database

Access to Atlas is available to participating members of the research networks supported by SCHARP as part of the Enterprise (CAVD, CHAVI, MTN, HVTN and VISC at present). To inquire about access, contact atlas@scharp.org. Published results and information about certain projects are available to the public without logon at https://atlas.scharp.org. For example, all completed CAVD studies are published on Atlas in the VISC folder.

## Abbreviations

**AIDS**: Acquired immune deficiency syndrome; **API**: Application programming interface; **CAVD**: the Collaboration for AIDS Vaccine Discovery; **CHAVI**: the Center for HIV Vaccine Immunology; **the Enterprise**: the Global HIV Enterprise; **HIV**: Human immunodeficiency virus; **HTML**: Hypertext markup language; **HVTN**: the HIV Vaccine Trials Network; **LDAP**: Lightweight directory access protocol; **LDMS**: Laboratory Data Management System; **LIMS**: Laboratory information management system; **MTN**: the Mircrobicide Trials Network; **NAb assay**: Neutralizing antibody assay; **SCHARP**: the Statistical Center for HIV/AIDS Research & Prevention at the Fred Hutchinson Cancer Research Center; **SQL**: Structured query language; **SSO**: Single sign-on; **URI**: Uniform resource identifier; **VISC**: the Vaccine Immunology Statistical Center

## Authors' contributions

All LabKey Software authors have contributed design, implementation and documentation to LabKey Server. MI leads design of the LabKey platform. BP and SR lead and coordinate work across LabKey and Atlas teams, respectively. JE leads the LabKey development team and BP did so previously. EKN wrote most of this paper and leads LabKey documentation. CN develops widely-used applications on Atlas. TS contributed strategic vision and leadership. All authors contribute testing and have reviewed this paper.
